# Ostraceous psoriasis in end-stage renal disease: A delayed phenotype switch from dupilumab treated with guselkumab

**DOI:** 10.1016/j.jdcr.2026.05.049

**Published:** 2026-05-27

**Authors:** Andrew E. Craver, Paige McKenzie, Ailish Hanly, Matthew D. Vesely

**Affiliations:** a Yale School of Medicine, New Haven, Connecticut; b Department of Dermatology, Yale School of Medicine, New Haven, Connecticut

**Keywords:** atopic dermatitis, cytokine switch, dupilumab, end-stage renal disease, guselkumab, IL-4/IL-13 blockade, ostraceous psoriasis, paradoxical psoriasis, phenotype switch, rupioid psoriasis

## Introduction

Dupilumab, a fully human monoclonal antibody targeting the interleukin (IL)-4 receptor α subunit, inhibits IL-4 and IL-13 signaling and has become a mainstay treatment for moderate-to-severe atopic dermatitis (AD).^1^ By suppressing type 2 inflammation, dupilumab improves pruritus, sleep, and skin barrier integrity.^2^^,^^3^ However, a growing body of literature describes “paradoxical” inflammatory eruptions emerging during IL-4/IL-13 blockade, including psoriasis and psoriasiform dermatitis.^4^^,^^5^ This phenomenon is thought to reflect immune repolarization toward T helper (Th) 17/IL-23 pathways after suppression of Th2 inflammation, representing a “phenotype switch” rather than a true “paradoxical” eruption.^1^^,^^4^^,^^6^

Here, we describe a patient with end-stage renal disease (ESRD) and chronic eczematous dermatitis who developed ostraceous psoriasis after long-term dupilumab therapy, confirmed histologically and by cytokine profiling. The eruption resolved completely following transition to IL-23 inhibitor guselkumab.

## Case presentation

**Fig 1:**
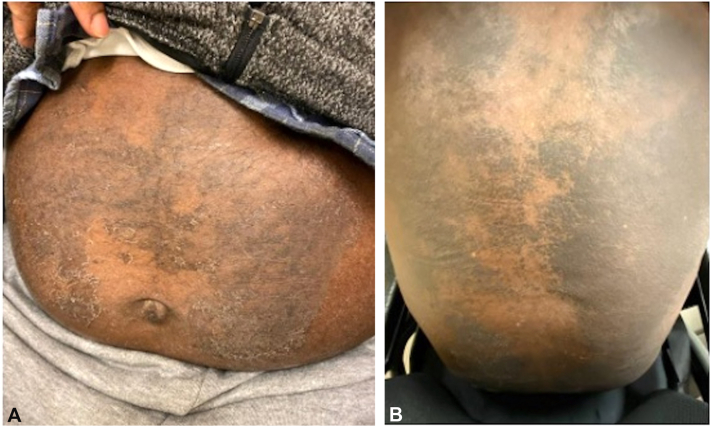
Clinical images of eczematous dermatitis. Hyperpigmented, lichenified, scaly plaques on abdomen **(A)** and back **(B)**.

**Fig 2:**
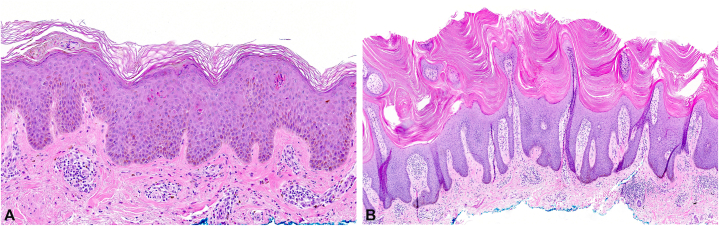
Histopathology of biopsies. Histological evaluation of eczematous rash left forearm shows spongiosis and perivascular infiltrate **(A)**. Histological evaluation of ostraceous plaque on right forearm shows acanthosis, parakeratosis with neutrophilic aggregates, hypogranulosis, and dilated dermal vessels consistent with psoriasis **(B)**.

A 78-year-old man with ESRD on hemodialysis presented in August 2021 with severe generalized pruritus and a diffuse lichenified eruption (Fig 1) refractory to topical triamcinolone and low-dose gabapentin. Because of recalcitrant symptoms, dupilumab was initiated (600 mg subcutaneously once, then 300 mg every 2 weeks), resulting in substantial early improvement of pruritus and eczematous lesions. Within several months, the patient developed partial recurrence of thickened plaques on the extremities. Bilateral arm biopsies demonstrated chronic spongiotic dermatitis with necrotic keratinocytes (Fig 2, *A*), consistent with eczema. Differential diagnosis of scabies and cutaneous T-cell lymphoma were considered and intradepartmental dermatopathology review at the time of biopsies was performed and consensus favored an eczematous process. A short prednisone taper (20 mg daily for 14 days) and adjunctive topical halobetasol, followed later by ruxolitinib 1.5% cream, yielded gradual improvement and allowed tapering of gabapentin. He remained itch-free and clear of rash on dupilumab monotherapy.

**Fig 3:**
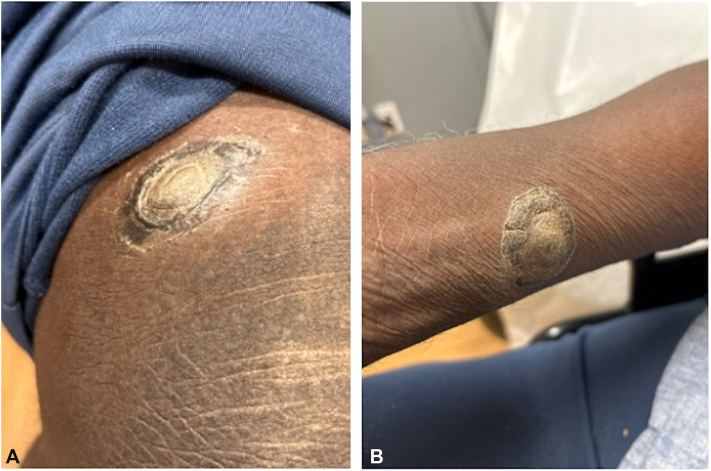
Clinical images of ostraceous psoriasis. Oval to round plaques with thick, adherent scale with concave center on left knee **(A)** and cone-like scaling on right forearm **(B)**.

**Fig 4:**
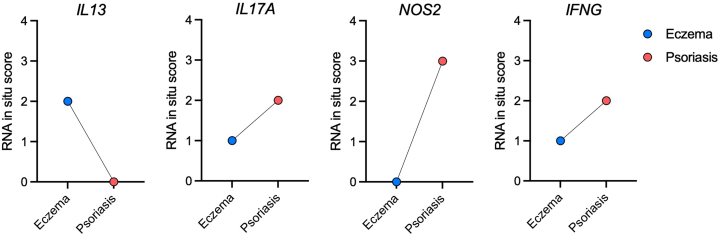
RNA in situ analysis for cytokines. Skin biopsies performed for diagnosis from eczematous eruption and ostraceous psoriasis were stained with RNA probes for *IL13*, *IL17A*, *NOS2*, and *IFNG*.

After nearly 3 years of stable disease control on dupilumab, new rapidly enlarging oval-to-round hyperkeratotic plaques with adherent concentric scale on the arms and legs (Fig 3). Biopsy revealed acanthosis, parakeratosis with neutrophilic aggregates, hypogranulosis, and dilated dermal vessels (Fig 2, *B*). RNA in situ hybridization demonstrated strong IL-17A and nitric oxide synthase 2 (NOS2) positivity with negligible IL-13 staining, diagnostic of psoriasis vulgaris, ostraceous type (Fig 4). This is contrast to previous spongiotic biopsy, which showed IL-13 positivity, mild IL-17A staining, and NOS2 negative, consistent with mixed Th2/Th17 polarization (cytokine staining was performed retrospectively for comparison) (Fig 4).

High-potency topical corticosteroids were prescribed, producing only partial improvement. Within weeks, additional psoriatic plaques developed on the scalp and trunk. Dupilumab was discontinued and guselkumab 100 mg subcutaneously every 8 wk (after a loading dose at week 4) was initiated, with rapid improvement. At 2 months, the rash was resolving and at 9 months all psoriatic plaques had resolved, leaving post-inflammatory hyperpigmentation only. The patient remained clear and asymptomatic after over a year of ongoing guselkumab maintenance therapy.

## Discussion

Paradoxical cutaneous reactions to targeted biologics are increasingly recognized.^5^ Rather than representing a truly ‘paradoxical’ reaction, these cutaneous eruptions are increasingly understood as an immunophenotypic switch resulting from the disruption of cytokine balance. Under normal conditions, IL-4 and IL-13 exert a regulatory ‘brake’ on the Th17/IL-23 axis; blocking the IL-4Rα subunit with dupilumab can release this inhibition, permitting a drift toward Th17-dominant inflammation. Our patient’s 3-year latency period demonstrates that this switch can occur even after prolonged Th2 suppression, a finding supported by our RNA in situ hybridization showing robust IL-17A and NOS2 expression with negligible IL-13 (Fig 4).

Dupilumab-associated psoriasis is now a well-documented and increasingly reported phenomenon, often arising within months of therapy initiation in patients treated for AD or prurigo nodularis.^1^^,^^4^ The mean time to onset reported by Brumfiel et al was 3.7 months, with about half of cases requiring discontinuation of dupilumab.^4^ Our patient’s latency of about 3 years suggests that delayed immune drift can occur even after long-term stability.

Management strategies for this phenotype switch depend on severity. Many cases improve with topical therapy, including corticosteroids, though approximately 50% of patients ultimately require dupilumab discontinuation.^1^^,^^4^ Therapeutic escalation follows psoriasis treatment paradigms, with literature review of published reviews and case reports describing improvement or resolution after transitioning totargeted agents including IL-12/23 inhibitors (ustekinumab), IL-23 inhibitors (guselkumab, risankizumab), and JAK inhibitors (upadacitinib, baricitinib).^1^^,^^7^^,^^8^ Combination therapy of dupilumab and guselkumab demonstrated improvement in both AD and psoriasis in 83% of patients in a small series.^1^ Other combinations including dupilumab with secukinumab or adalimumab have also been reported without significant safety signals, though long-term monitoring remains limited.^1^ Targeting TYK2 with deucravacitinib represents a mechanistically rational option, but is understudied in dupilumab-associated psoriasis.

The sustained absence of eczematous relapse following the transition to guselkumab suggests that IL-23/Th17 signaling likely contributed to the patient's baseline dermatitis. This is supported by our initial cytokine profiling, which demonstrated a mixed Th2/Th17 signature with baseline IL-13 and IL-17A positivity prior to the overt clinical transition to psoriasis (Fig 4). This suggests an immunophenotypic switch where the underlying inflammatory landscape became progressively Th17-dominant under chronic IL-4/IL-13 blockade. By targeting the IL-23 pathway, guselkumab likely suppressed both the emergent ostraceous psoriasis and the Th17 component of the original eczematous disease, maintaining long-term clinical clearance. For patients with comorbid ESRD, the selection of an IL-23 inhibitor like guselkumab is particularly advantageous, as it avoids the renal toxicity associated with conventional systemic agents like methotrexate or cyclosporine and has demonstrated more favorable renal safety profiles.^9^

This case expands the spectrum of dupilumab-associated psoriasis by highlighting an ostraceous variant emerging after prolonged therapy in a medically complex patient. Morphologically, some lesions had concave-centered plaques resembling oyster shells (ostraceous) while other plaques were characterized by cone-shaped limpet-like lesions, sometimes referred to as rupioid psoriasis. We consider both ostraceous and rupioid variants to be on a clinical continuum of exuberant hyperkeratosis. Our case extends the latency of phenotypic shift that may occur on biologic therapy and highlights a unique clinical scenario of a patient with comorbid ESRD and appropriate treatment selection. Clinicians should consider psoriasis in any new hyperkeratotic eruption during IL-4/IL-13 blockade, even after years of control. Cytokine immunohistochemistry can aid diagnosis and guide rational biologic selection.^10^

## Conflicts of interest

None disclosed.
